# Applying concepts from “rapid” and “agile” implementation to advance implementation research

**DOI:** 10.1186/s43058-022-00366-3

**Published:** 2022-11-05

**Authors:** Andrew Quanbeck, Rose Garza Hennessy, Linda Park

**Affiliations:** 1 Department of Family Medicine and Community Health, University of Wisconsin-Madison, 800 University Bay Drive, Madison, WI 53705 USA; 2 Joseph J. Zilber School of Public Health, University of Wisconsin-Milwaukee, P.O. Box 413, Milwaukee, WI 53205 USA

**Keywords:** Agile implementation, Rapid implementation, Systems engineering, Behavioral economics, Complex systems

## Abstract

**Background:**

The translation of research findings into practice can be improved to maximize benefits more quickly and with greater flexibility. To expedite translation, researchers have developed innovative approaches to implementation branded as “rapid” and “agile” implementation. Rapid implementation has roots in precision medicine and agile implementation has roots in systems engineering and software design. Research has shown that innovation often derives from learning and applying ideas that have impacted other fields.

**Implications for implementation researchers:**

This commentary examines “rapid” and “agile” approaches to implementation and provides recommendations to implementation researchers stemming from these approaches. Four key ideas are synthesized that may be broadly applicable to implementation research, including (1) adopting a problem orientation, (2) applying lessons from behavioral economics, (3) using adaptive study designs and adaptive interventions, and (4) using multi-level models to guide implementation. Examples are highlighted from the field where researchers are applying these key ideas to illustrate their potential impact.

**Conclusions:**

“Rapid” and “agile” implementation approaches to implementation stem from diverse fields. Elements of these approaches show potential for advancing implementation research, although adopting them may entail shifting scientific norms in the field.

Contributions to the literature

- “Rapid” and “agile” implementation approaches are innovative methods to counteract the perceived slowness and ineffectiveness that characterizes the translation of research findings into practice.
- Four main principles are presented from “rapid” and “agile” implementation that may be broadly applicable to implementation research such as adopting a problem orientation, using lessons from behavioral economics, being adaptive in both design and intervention, and guiding implementation using multi-level models.
- Although the ideas advocated for by rapid and agile approaches to implementation have proven useful in other fields and may hold promise for implementation science, more work is needed to fully embrace them within this field.

## Background

The perceived slowness with which research findings translate into practice has been a longstanding source of frustration for stakeholders across the implementation research domain [1]. Implementation researchers are familiar with the statistics stating how it takes 17 years, on average, for a handful of research findings to be adopted in clinical practice [2]. The 12th Academy Health/National Institutes of Health annual conference on Dissemination and Implementation Research (held in Washington, D.C., between December 4 and 6, 2019) included “rapidity” as a theme and had a plenary session on “rapid, relevant, and rigorous research” that attempted, in part, to address this familiar time lag. In the realm of drug development, the time from drug discovery to clinical testing and approval can take up to 15 years [3]. Accurate measurement of the time required for translation is challenging, given the vast range of potential interventions and implementation targets. In any case, there is widespread agreement that knowledge translation takes too long, in particular from the perspective of clinicians who need rapid, actionable data to make treatment decisions [1].

Beyond the problem of slowness, the field lacks basic information on how to implement interventions *effectively.* The Tailored Implementation in Chronic Diseases (TICD) project [4] was perhaps the most expansive implementation trial conducted worldwide, spanning 5 countries (Germany, Netherlands, Norway, Poland, and the UK) and 5 chronic diseases (multimorbidity, vascular conditions, depression, chronic obstructive pulmonary disease, and obesity). The study was guided by theoretical work suggesting that adaptation of interventions and implementation strategies is needed to operate within the complex contexts in which implementation occurs [5]. Across the 5 studies, group interview methods with healthcare providers and other stakeholders produced more than 1200 potential determinants upon which to tailor implementation approaches [6], including more than 600 determinants that were deemed valid by health professionals involved with implementation [7]. Overall, the tailored implementation programs developed in the TICD project showed little evidence of effectiveness. However, the findings pointed to potential areas for further research, including calls to experiment with different sampling and interview methods and the need to systematically prioritize determinants and potential adaptations based on a large number of candidates [8].

### Rapid implementation

To counteract the perceived slowness with which translation occurs, the topic of “rapid implementation” has begun gaining currency among implementation scientists. A group of Australian and UK-based researchers led by Dr. James Smith from Macquarie University [1] point out that many existing definitions of implementation science and popular implementation models lack an explicit temporal dimension. This is potentially problematic—Everett Rogers’ classic text *Diffusion of Innovations (1995)*, foundational to the field of implementation science, makes it clear that *time is a fundamental element* in the diffusion process, and as such cannot be ignored [9]. Smith et al. [1] conducted a systematic integrative review of 24 articles to identify works that related to rapid implementation and used concept analysis to define the term explicitly. Their process resulted in this definition:

Rapid implementation provides the best possible evidence-based practice of a program or intervention to those who need it, with speed and efficiency, by redefining rigour, and adapting both methods (adapting current approaches, procedures and implementation frameworks), and trial design, to fit research aims and objectives….The theoretical definition derived from our results characterises rapid implementation as incorporating speed and efficiency, while having the ability to adapt methods and trial design to suit the needs of complex studies.

The concept of rapid implementation emerged primarily from the domain of precision medicine. Rapid implementation seeks to adapt and extend concepts that have become influential in precision medicine to implementation research. Among several recommendations, updates to the efficiency of clinical trial designs are noted as a promising area for improvement; in particular, the authors recommend greater use of adaptive trial designs, such as basket or umbrella trials. These types of study designs rely on Bayesian decision rules to determine whether treatments with low probabilities of success should be discontinued and therapies with high probabilities of future success should advance [10]. Smith et al. [1] acknowledge that the literature on rapid implementation is still largely conceptual and lacking in empirical results as of 2020.

### Agile implementation

In recent years, the concept of *agile implementation* has been advanced by Boustani and colleagues from the University of Indiana in the USA [11, 12], providing another set of innovative ideas to potentially guide implementation research. Agile implementation rests on several core ideas. First, implementation is posited to occur in the context of complex adaptive systems, defined as networks of semiautonomous individuals who are interdependent and connected in multiple nonlinear ways [12]. The agile implementation model invokes Chaudoir et al.’s [13] multi-level implementation framework. This framework depicts five macro–micro level nested factors that influence the implementation of innovations, including structural-, organizational-, provider-, patient-, and innovation-level measures. Agile implementation also explicitly incorporates behavioral economic factors by describing human tendencies in information processing and decision-making that can be used to modify the social and physical environment to promote successful implementation. The agile implementation model has been used to reduce central line–associated bloodstream infections in hospital settings [14] and to reduce dementia symptoms in a safety net healthcare delivery system [11].

Prior to application within implementation research, the term “agile” has arguably had the most purchase in the realm of software development. The Agile Manifesto [15], published in 2001 during a conference attended by 17 leading software developers, embraced a set of principles that included (1) valuing customer satisfaction (above all else), (2) responding to change vs. sticking with a plan that is not working, and (3) always striving to reduce the time to delivery of working software. A primary distinction between the literature on “rapid” and “agile” implementation relates to application areas, where rapid implementation has been associated with precision medicine (related to drug development for cancer), while agile implementation has been applied in clinical (hospital and primary care) settings.

## Implications for implementation researchers

What might the implementation science field learn from ideas originating from other fields, like precision medicine and software development? The US National Academy of Engineering defines engineering as fundamentally “making science useful to people” [16]. Systems engineers understand that innovation often stems from understanding a wide array of disciplines, through synthesizing key ideas from diverse fields and applying them pragmatically to the problem at hand. Indeed, research on organizational change shows that most innovative ideas come from other fields [17]. Lessons from outside the field are potentially limitless, but this paper will provide a rationale for expanding the use of several approaches suggested by rapid and agile approaches that may hold the potential to advance the field. We propose that implementation scientists can enhance their ability to engage in rapid and agile implementation by drawing upon methodological approaches from other disciplines that have centered their work around these aims (i.e., systems engineering, precision medicine, etc.). We offer four major considerations for implementation scientists, not as a prescriptive or fully exhaustive list, but as a manner of synthesizing literature outside the field of implementation science.

Before reviewing these considerations, it is essential to acknowledge the challenges inherent to rapid implementation. Despite the urgency to increase the timeliness of implementation, rapid implementation can cause unintended consequences. Proctor et al. [18] explore the trade-offs in prioritizing implementation speed and explore the argument that “implementation occurs too slowly, versus implementation should not be rushed.” Since rapid implementation *redefines rigor*, there is the possibility that innovations implemented may be more likely than others to require de-implementation, meeting the criteria established by McKay et al. [19] in being ineffective or harmful, not the most effective or efficient, or no longer necessary.

Tension for change is a necessary condition preceding successful implementation, and one that can at least ensure that innovation is called for [20, 21]. However, creating tension for change is difficult, suggesting that implementation researchers should focus on rapid implementation to address problems for which tension for change already exists. Rapid implementation may not be desirable without sufficient tension for change and in some cases may trigger the subsequent need for de-implementation [22]. Furthermore, focusing solely on rapid approaches may allow for less time to elicit multiple and diverse perspectives to inform the implementation process and could even exacerbate inequities and health disparities, as demonstrated in previous implementation research [23, 24].

Despite the challenges inherent to rapid implementation, we agree with Proctor et al. [18] that the public health challenges of our time *demand* timely implementation. Even in cases where de-implementation may be later required (which are not limited to innovations that are rapidly implemented), mechanisms used in rapid implementation may also assist to more rapidly engage in de-implementation. This paper adds to the literature to assist implementation scientists to expand the mechanisms used to redefine rigor among stakeholders, to ensure that rigor is not disregarded and diverse end-users are not neglected, but instead that rapid and agile approaches can be applied to be more responsive in an expedient and equitable manner.

### Adopting a problem orientation

Adopting a problem orientation in a proactive and strategic manner is important for rapid and agile implementation because (1) it helps to identify problems that are more likely to have urgency, which increases the likelihood of stakeholder buy-in to more quickly and flexibly identify and implement health innovations in prioritized areas; (2) using strategic planning techniques can help researchers gather feedback from multiple stakeholders in a rapid manner, increasing the timeliness of implementation while still providing the opportunity for diverse input; and (3) multi-stakeholder engagement with facilitation techniques that aim to gather and incorporate feedback from all participants, regardless of status within a group, may also increase health equity by designing and tailoring implementation strategies and connecting to systems and sectors outside of health, which are recommendations by Brownson et al. [24] to increase health equity in implementation science.

Adaptive or tailored approaches to implementation strategy design and intervention delivery are increasingly recognized as essential [4]. One notable feature of the TICD study was its widescale use of open-ended brainstorming with stakeholders. It is critical to emphasize the importance of stakeholder engagement in implementation research, and with good reason. Consistent with the first principle of the Agile Manifesto, research from the field of systems engineering indicates that understanding and involving the customer perspective is the single most important factor in achieving organizational change [18]. An engineering designer comes in with questions for customers—not answers—and begins with understanding the problem, not presupposing the solution. Important stakeholders in implementation research may include patients, clinicians, organizational and community leaders, and payers. Stakeholders can be identified in multiple ways, from informally referencing organizational charts to more complex social network analyses; numerous models and frameworks exist to support this work [25, 26]. It is essential to understand what matters to different groups of stakeholders and to learn how to prioritize implementation projects within stakeholders’ existing value systems. One viable and simple place approach to problem exploration from the domain of engineering is the model for improvement [27]. The model for improvement has been used in countless applications outside of healthcare dating back to the 1970s, with Japan’s Toyota Motor Company exemplifying its use and impact on quality in manufacturing. The model for improvement has also been adapted for use in healthcare and is foundational to an organizational change model that has been widely employed across addiction and behavioral health providers in the USA [28, 29]. It is built on three simple questions:

1. What are we trying to accomplish?
2. How will we know if a change is an improvement?
3. What change(s) can we try?

These elegantly simple questions can be very productive when asked of the various stakeholders involved in a decision to make a change (like adopting a new health intervention). These types of questions are meant to be conversational and bidirectional, used as a means of inquiry with customers, broadly defined.

The nominal group technique [30] is a facilitation technique that provides a useful way to conduct an inquiry related to the model for improvement, including explicit steps for identifying the problem, generating solutions, and prioritizing ideas to implement through a group voting process. This technique can be done with multiple stakeholders and completed within an hour or two, expediting the prioritization process. It is the implementation researcher’s job to identify the right stakeholders to bring to the table and pose the right questions for planning purposes. Asking these types of questions using the nominal group technique is a simple and well-established way to manage the process of stakeholder engagement so critical to implementation research. The open-ended methods of inquiry (brainstorming, etc.) used in the TICD study [8] are very much in line with the spirit of the model for improvement. Notably, the nominal group technique contains an explicit step for prioritizing ideas generated through brainstorming that could respond to the recommendations coming out of the TICD project.

In the domain of primary care, Miller et al. [31] have suggested the need to shift Dissemination and Implementation (D&I) research from the model of a scientist approaching a clinic with a specific practice for implementation (outside-in approach) to a model that empowers clinicians to determine their own priorities and request D&I assistance in accordance with the needs that best serve their patients (inside-out approach). Etz et al. [32] further suggest that a thorough understanding of what provides value in primary care from the perspectives of patients, clinicians, and healthcare payers is essential to contextualizing and prioritizing implementation research proposals. Planning in advance before adopting a problem orientation with such stakeholders can allow for the prioritization process to happen more efficiently, rapidly, and equitably. If a researcher does not start with curiosity about what people in a given system actually want and need, implementation typically would not work—defense mechanisms activate, and human beings have hundreds of these decision-making defense mechanisms at their disposal [33]. Much of what is known about maximizing expected value from various perspectives comes from research in decision analysis and behavioral economics.

### Applying lessons from behavioral economics

Agile implementation stresses the importance of behavioral economics [12]. One of the canonical works of the behavioral economic school is Herbert Simon’s [34] classic book *Administrative Behavior*. In it, Simon states:

It is impossible for the behavior of a single, isolated individual to reach any high degree of rationality.

Decision-makers act rationally to a degree, but their decision-making is bounded by cognitive limitations, constraints in the environment, and limited time to make decisions. Examples of two bounding constraints on rational decision-making include (1) the impracticality of fully considering all the possible alternatives any one individual must explore to make a fully informed, rational decision and (2) time limits on decision-making. People operate in the context of organizations, which can be conceptualized as formalized decision-making structures where the focus is on a common purpose. Organizations place their members in a psychological environment that adapts their decision-making preferences to organizational objectives and imposes decision-making constraints that organizational members operate within.

Most people make decisions based on the information available (bounded rationality) as opposed to investigating all possible choices (perfectly rational). Pragmatically speaking, implementation researchers typically deal with what Simon referred to as “administrative man,” who makes bounded decisions that are satisficing, with a nearly universal preference for inaction (unless compelled otherwise). The status quo bias is a powerful conservative force that must be recognized and reckoned with in implementation research [33]. To run a successful implementation project, an essential first step is to engage the players whose input and coordination will be required for implementation to occur and get them to acknowledge that they are part of a dynamic process that requires decision-making. In his groundbreaking research in behavioral economics, Daniel Kahneman conceptualizes two modes of human decision-making: system 1 and system 2. System 1 is the reflexive, unconscious kind of thinking that characterizes human cognition the vast majority of the time. System 2 is the reflective and conscious decision-making process that academicians assume that humans engage in as a matter of course (which, as countless empirical findings from behavioral science tell us, they do not) [26].

If actors whose engagement in the implementation process are not aware that an implementation process is going on, they are not making any decisions and evidence-based practices are not getting adopted. In the absence of systematic efforts on the part of implementation researchers, system 1 thinking predominates, and that type of unconscious thinking is a recipe for maintaining the status quo. This can be especially true when researchers overlook differing perspectives that may predominate in racially and ethnically diverse populations and fail to represent these perspectives as part of the research team. Strategies need to be put in place to engage system 2. Conscious effort needs to go into convening the players and getting them engaged in a conscious decision-making process.

Even if a change agent can convene the key stakeholders and get them to acknowledge that an active process of implementation is taking place that requires action on their part, the players still need to make the choices that the implementation researcher needs them to make—that is, to adopt the new practice (i.e., to cooperate, using the lexicon of game theory). The implementation researcher still has a host of vexing decision-making biases to overcome [33]. Researchers like Piderit [35] and Ford et al. [36] have reconceptualized resistance to change not as negative (the view often held by external change agents) but instead as ambivalence, and a resource to be sought out and used to develop or improve a potential change. Those who want to implement interventions must involve all relevant stakeholders, in part by actively listening to, explaining, and persuading adopters about the value of a potential change, a process that might benefit from using well-known management and marketing techniques on the “science of persuasion” [37].

The implementation researcher’s responsibility is to orchestrate an implementation strategy that actively seeks to understand and incorporate the objectives, resources, and constraints that drive decision-making among each group of essential stakeholders whose cooperation implementation success depends upon. The behavioral theories and frameworks driving agile implementation can facilitate this process [12]. The outcome that corresponds to successful implementation requires serial cooperation by each stakeholder group across multiple levels—a sort of decision-making cascade that only manifests when the perceived net benefit of adoption is sufficiently positive for each group to compel a positive adoption decision [38]. The decision to adopt, therefore, must represent a clear and sequential win–win-win–win scenario from the perspectives of payers, management, staff, and patients; failure at any level will lead to failure in total.

Beidas and colleagues at the University of Pennsylvania are doing exciting research at the nexus of implementation science and behavioral economics. Their research calls into question the assumption of rational decision-making in implementation science, using the rich literature of behavioral economics to identify and leverage cognitive heuristics to maximize the likelihood of desired behavior and identify ways to structure the environment to make it easier for clinicians to adopt evidence-based practices [39]. They have published the study protocols from their research center funded by the US National Institute of Mental Health on the application of behavioral economic concepts in healthcare, which describes an innovation tournament they designed to crowd-source ideas, methods for using behavioral insights to design implementation strategies, and a trial to use behavioral insights to increase patient adherence to taking selective serotonin reuptake inhibitors [40].

### Adaptive study designs and adaptive interventions

Smith et al. (2020) posed a challenge for the field to redefine rigor in implementation research by advocating for novel approaches for the conduct and design of implementation research studies in order to conduct rapid implementation [1]. In particular, they advocate for study design and analysis methods that are fundamentally Bayesian rather than frequentist in orientation. In advocating for more sophisticated study designs in precision medicine, cancer statistician Donald Berry stated [41]:

It is ironic that we take the same clinical trial approach to evaluate all manner of potentially amazing transformative experimental therapies and yet we don’t experiment with the design of the clinical trial itself.

Adaptive study designs can make clinical trials more flexible, efficient, and expedient as they allow for real-time modifications to a study or procedure while still adhering to high levels of rigor, advancing rapid and agile implementation [42]. One concept that is gaining traction in implementation research is the use of sequential, multiple-assignment randomized trials (SMARTs). While not technically adaptive study designs, SMARTs are examples of study designs that feature *adaptive interventions*. SMARTs were originally used to develop dynamic treatment regimens for mental health. The inventor of SMART trials, Dr. Susan Murphy, uses the analogy of treating a patient with alcohol use disorder in laying out the rationale for adaptive interventions [43]: A clinician might try a variety of different evidence-based treatments in creating a patient’s treatment regimen. For instance, the clinician might start with motivational interviewing, see how the patient does for a pre-specified monitoring period, and then readjust treatment plans if the patient is not responding to treatment. In that case, the therapist might add a medication like naltrexone and continue monitoring until symptoms improve, layering on more treatments until desired results are achieved. Similarly, SMARTs layer new components of interventions or additional implementation strategies in a strategic manner to maximize desired outcomes defined in advance by the experimenter. This study design is inherently agile, as it allows for flexibility and responsiveness as more information about a subject is learned, which also draws upon a Bayesian approach to tailoring implementation and analysis as more data becomes available. In the long run, SMART designs may also enhance rapid implementation, because a single study can utilize a variety of implementation strategies and approaches that may more quickly achieve implementation outcomes, instead of having to utilize only one approach at a time. The findings from a SMART may speed up the general knowledge of what works under which conditions, which again could assist in more rapidly building the evidence itself upon which to make implementation decisions. The basic idea of providing adaptive treatments based on monitoring a response variable has extended to implementation trials, where the research participants are typically clinicians or clinics that treat “clusters” of patients. The Adaptive Implementation of Effective Programs Trial was a groundbreaking clustered SMART focused on improving the treatment of mood disorders in community settings [44]. Replicating Effective Programs (REP) is a proven low-intensity implementation strategy designed to standardize the implementation of interventions into routine care through toolkit development and marketing, clinician training, and program assistance. The focal intervention in the trial was Life Goals, an evidence-based treatment for mood disorders delivered in six individual or group sessions. The trial, conducted in 80 community mental health and primary care clinics, used REP as a core implementation strategy for all sites. The study design allowed for systematic layering of additional implementation support based on monitoring of a response variable indicative of implementation success. The study design aimed to determine, among clinics that had been non-responsive to REP, the cost-effectiveness of adding external or internal facilitation as supplemental implementation strategies. A cost-effectiveness analysis found that the adaptive strategy that begins with a less intensive, less costly strategy, and increases implementation support based on monitoring the implementation response variable, was the most cost-effective strategy among those tested in the trial [45]. Resources such as those available at the NIH-funded Methodology Center at Pennsylvania State University [46] can help implementation researchers design studies that are both statistically rigorous and adaptive in their approach.

Adaptive study designs and adaptive interventions introduce another fundamental tension to implementation science. The more active role that response monitoring implies is contrary (in particular) to the concept of blinding. Effective monitoring systems used to adapt interventions are specifically designed to provide feedback, which is antithetical to the concept of single- and double-blinding in clinical trial design. Conceptually, traditional frequentist study designs sacrifice time and efficiency in the process. Bayesian thinking, on the other hand, implicitly involves continuous updating of prior beliefs based on new observations and adapting interventions accordingly. Said another way, the frequentist mindset demands experimenting with eyes closed (double blinded), whereas the Bayesian experimenter operates with eyes open (open label).

### Use multi-level models to guide implementation research

Multi-level models allow implementation scientists to study complexity, which is a guiding theory of agile implementation [12]. We acknowledge that multi-level models do not inherently make implementation more rapid, but that they increase the likelihood of success since context can be considered across levels of influence. Actively seeking to understand the perspectives and values of stakeholders across multiple levels—including patients, clinicians, organizational and community leaders, and payers—is essential to maximizing the perceived value of an innovation and raising the probability of implementation success [47]. Stakeholders’ values differ based on their unique vantage points on the system within which they are operating [38]. While the concept of multi-level conceptualization is not novel to the field, the consistent application of multi-level models within implementation theory, practice, and research would benefit from additional development. For example, the most commonly cited model in implementation science, the Consolidated Framework for Implementation Research (CFIR) [48], was compiled from previous literature and does not directly articulate hierarchical levels of influence. From a measurement standpoint, a systematic review found that fewer measures were available for structural and patient-level constructs, which represent individual and macro levels vital to achieve change; further, almost half of measures across all levels lacked criterion validity [13].

What exactly is a “system?” Herbert Simon, in addition to his foundational research in behavioral economics, has been an enormously influential thinker on systems analysis and a pioneer in the development of artificial intelligence. His classic paper *The Architecture of Complexity* [49] includes the following passage:

… complexity frequently takes the form of hierarchy, and . . . hierarchic systems have some common properties that are independent of their specific content. Hierarchy, I shall argue, is one of the central structural schemes that the architect of complexity uses…. By a hierarchic system, or hierarchy, I mean a system that is composed of interrelated subsystems, each of the latter being, in turn, hierarchic in structure until we reach some lowest level of elementary subsystem.

In the case of implementation research, the elementary subsystem is represented by individual people. Building an accurate representation of the complex, multi-level nature of the system to be intervened upon necessary precursor to successful agile implementation. Ferlie and Shortell’s [50] multi-level model of system change may be used in implementation research which focuses primarily on healthcare organizations and clinicians. Many other robust multi-level models exist to acknowledge and account for the complexity and therefore implement in a flexible and agile manner. The socio-ecological framework [51] has nested levels of relationships (including individuals, families, communities, and society at large) that may be especially appropriate for application in community-based implementation research.

Identifying the relevant stakeholders in a system is a first step in an implementation research project. Organizational charts can be helpful as a starting point in mapping out the actors in a system and the relationships between them. However, such static representations of system architecture tend to be limited in their usefulness in conducting research. Implementers need to talk with stakeholders within the organization and the community to understand how decisions related to implementation are really made.

Rittel and Weber’s [52] discussion of wicked problems makes the relevance of multi-level models clear in another way. In brief, a wicked problem arises in part because different stakeholder groups within a system have different values, and these values may not align. Rittel and Weber write:

Our point, rather, is that diverse values are held by different groups of individuals—that what satisfies one may be abhorrent to another, that what comprises problem–solution for one is problem-generation for another. Under such circumstances, and in the absence of an overriding social theory or an overriding social ethic, there is no gainsaying which group is right and which should have its ends served.

Some of our own research on opioid prescribing relates to the discussion of wicked problems [38]. In 2016, the US Centers for Disease Control issued clinical guidelines for opioid prescribing [53] to help mitigate the ongoing opioid crisis in the USA. These guidelines recommend (among other things) a dosing limit of 90 morphine-milligram equivalent units to reduce the risk of opioid overdose. Different stakeholders in the system might see this problem from vastly different perspectives. At the societal level, CDC wants these guidelines implemented for public safety, and health systems leaders want to fall in line with CDC recommendations, often issuing organizational policies based upon them. Patients can end up on high opioid doses to treat legitimate pain because their primary care physician has prescribed high doses for years. Some state regulators have taken legal action against primary care physicians for over-prescribing opioids. In some of these cases, clinicians can solve a problem (from their perspective) by terminating clinician-patient relationships with patients on high-dose opioids. Terminated patients may then turn to illicit heroin or fentanyl to deal with opioid withdrawal and may subsequently overdose. The patient bears the brunt in this scenario, and society loses overall even though the clinician and the health system have “done their job” by reducing overall opioid use by their patients. An agile approach to implementation would acknowledge the multi-level complexity of opioid prescribing and consider implementation of guidelines that center patient needs alongside the needs of clinicians and health systems. Opioid prescribing presents wicked problems indeed. This scenario illustrates the need for multi-level models to at least try to understand and mitigate the potential for adverse outcomes if the system is circumscribed in ways that disadvantage particular stakeholders within it.

Zimmerman and colleagues at Stanford University have built comprehensive models of participatory system dynamics to increase timely patient access to evidence-based pharmacotherapy and evidence-based psychotherapy for depression, PTSD, and alcohol and opioid use disorders in the US Veterans’ Administration [54]. Their models are theoretically grounded in Herbert Simon’s behavioral economic concept of bounded rationality [34] and John Sterman’s concept of “double-loop learning” from systems science [55]. When it comes to modeling complex adaptive systems in implementation research, Zimmerman et al. have an extremely broad and deep corpus of research developed and under development. Readers can learn more by visiting their online resources on “Modeling to Learn” at http://mtl.how/demo.

## Conclusions

To conduct implementation studies that are informed by the paradigms of rapid and agile implementation, implementation researchers should first define the system they intend to intervene upon as accurately as possible. Systems are interconnected and hierarchically nested networks of persons across different levels. The implementation researcher then needs to learn the values of the key decision-makers in the system to determine how implementation research can meet their needs. Finally, the lessons of behavioral economics can inform researchers on how to frame decisions, present alternatives, and suggest strategies for optimizing the value of interventions to various stakeholder perspectives.

The field of implementation science continues to repeat the familiar, seemingly abysmal statistics (14%, 17 years) on the rate at which evidence-based practices are adopted [2] without recognizing this as a form of “base rate neglect,” as described by Kahneman [33]. Implementation is hard. By way of contrast, it is generally accepted that 90% of all business startups fail. While acknowledging that reliable statistics are scarce in the domain of business, should researchers and practitioners expect successful implementation to be anything less than difficult? The ideas advocated for by rapid and agile approaches to implementation have proven useful in other fields and embracing them more frequently in implementation research may help to close implementation gaps. It is encouraging to see implementation researchers independently synthesizing ideas from relevant fields of science and engineering in novel ways and converging on potential solutions about what it will take to maximize the usefulness of science to people.

## Data Availability

N/a.

## Abbreviations

REP: Replicating Effective Programs
SMART: Sequential, Multiple-assignment Randomized Trial
TICD: Tailored Implementation in Chronic Diseases
